# Metagenomics analysis of bacteriophages and antimicrobial resistance from global urban sewage

**DOI:** 10.1038/s41598-021-80990-6

**Published:** 2021-01-15

**Authors:** Josephine E. S. Strange, Pimlapas Leekitcharoenphon, Frederik Duus Møller, Frank M. Aarestrup

**Affiliations:** Research Group for Genomic Epidemiology, National Food Institute, Technical University of Denmark, Building 204, 2800 Kemitorvet, Kgs. Lyngby Denmark

**Keywords:** Antimicrobials, Bacteriophages

## Abstract

Bacteriophages, or phages, are ubiquitous bacterial and archaeal viruses with an estimated total global population of 10^31^. It is well-known that wherever there are bacteria, their phage counterparts will be found, aiding in shaping the bacterial population. The present study used metagenomic data from global influent sewage in 79 cities in 60 countries to identify phages associated with bacteria and to explore their potential role in antimicrobial resistance gene (ARG) dissemination. The reads were mapped to known databases for bacteriophages and their abundances determined and correlated to geographic origin and the countries socio-economic status, as well as the abundances of bacterial species and ARG. We found that some phages were not equally distributed on a global scale, but their distribution was rather dictated by region and the socioeconomic status of the specific countries. This study provides a preliminary insight into the global and regional distribution of phages and their potential impact on the transmission of ARGs between bacteria. Moreover, the findings may indicate that phages in sewage could have adopted a lytic lifestyle, meaning that most may not be associated with bacteria and instead may be widely distributed as free-living phages, which are known to persist longer in the environment than their hosts. In addition, a significant correlation between phages and ARGs was obtained, indicating that phages may play a role in ARG dissemination. However, further analyses are needed to establish the true relationship between phages and ARGs due to a low abundance of the phages identified.

## Introduction

Bacteriophages are ubiquitous bacterial and archaeal viruses, with an estimated total global population of more than 10^31^, surpassing that of bacteria (10^29^) making them the most abundant biological entities in the biosphere. Their genomes, ranging from 2.4 to 735 kb^1,2^, consist of either DNA or RNA which can be single-stranded (ss) or double-stranded (ds).

Phages can engage in horizontal gene transfer through either specialised or generalised transduction^3^. In general, transduction of antimicrobial resistance genes (ARGs) in clinical settings occurs at extremely low frequencies of approximately 10^–9^ and 10^–7^ transductants/pfu for specialised and generalised transduction, respectively^4^. The role of transduction for gene transfer within a clinical setting is well-established, and there is growing evidence that phages, through transduction, aid in the dissemination of ARGs in the environment^5–8^. However, this is still controversial as contrasting research also indicates that the number of phage-encoded ARGs is overestimated^9^.

The emerging antimicrobial resistance is one of the biggest health threats globally. A report from 2016^10^ estimated that the total number of global deaths from infectious disease caused by antimicrobial resistance was 700,000 and predicted to have risen to 10 million by 2050. Furthermore, antimicrobial resistance is expected to have a huge economic impact, with an estimated inaction cost of US$100 trillion between 2016 and 2050. Therefore, one of the key recommendations for the prevention of deaths attributed to antimicrobial resistant infectious disease and the resulting economic burden is an increased monitoring of ARGs in the environment.

Metagenomic shotgun analyses have become a popular means of achieving ARGs surveillance, and sewage has proven an enriched, anthropogenic, and ethical source of information, especially in monitoring pathogens and the spread of ARGs^11–13^. As many residual antimicrobials are detectable in urban sewage due to either inappropriate disposal or incomplete metabolism such as fluoroquinolones, which are excreted unchanged through urine and can eventually enter environment through waste water^14^. A selective pressure is exerted upon bacteria living under these sewage conditions, which in turn have the potential to activate latent prophages. Furthermore, free-living phages have been shown to persist in the environment for longer than their hosts^15,16^, which could potentially lead to the further spread of ARGs among bacteria.

A study published in 2019 by Hendriksen et al.^11^ found a systematic regional difference in the bacterial population and ARGs in global urban sewage. The study included 79 metagenomic samples from 60 different countries collected during 2016 and investigated the global distribution of ARGs. However, the study did not investigate the association between ARG distribution and phages. Therefore, the present study aimed to characterise the phages associated with bacteria in the metagenomics data in order to determine whether there was a corresponding regional segregation. For this purpose, some of the largest viral databases were explored. Furthermore, the occurrence and diversity of phages across the globe were examined to evaluate whether socioeconomic status influenced the distribution. Finally, a potential correlation between the observed phages and bacteria in addition to ARGs was assessed.

## Results

Most virus sequences obtained from the NCBI database were of bacteriophages; of 719 total accession numbers, 638 were bacteriophages while 81 were other viruses. Of these 81 other viruses, 7 were bacteriophages but were not properly annotated in NCBI and thus not captured. For the IMG/VR database, a total of 54,893 sequences were bacteriophages of which 3699 were more than 90% completed, high-quality draft genome or entirely completed genomes (the latter collectively referred to as “IMG/VR genomes”); 24,348 sequences were other viruses. A total of 114 bacteriophages from the huge phage database were obtained.

Mapping results showed that the mean coverage and depth of phages within the various databases were generally low (Table S1 and Fig S1 to Fig S3) with mean coverage and depth ranging between 1.21–2.5% and 0.03–0.18%, respectively, across databases. Due to the extremely low abundance of most phages (Fig S4), no phages were excluded based on the coverage and depth analysis.

### Mapping summary

**Figure 1:**
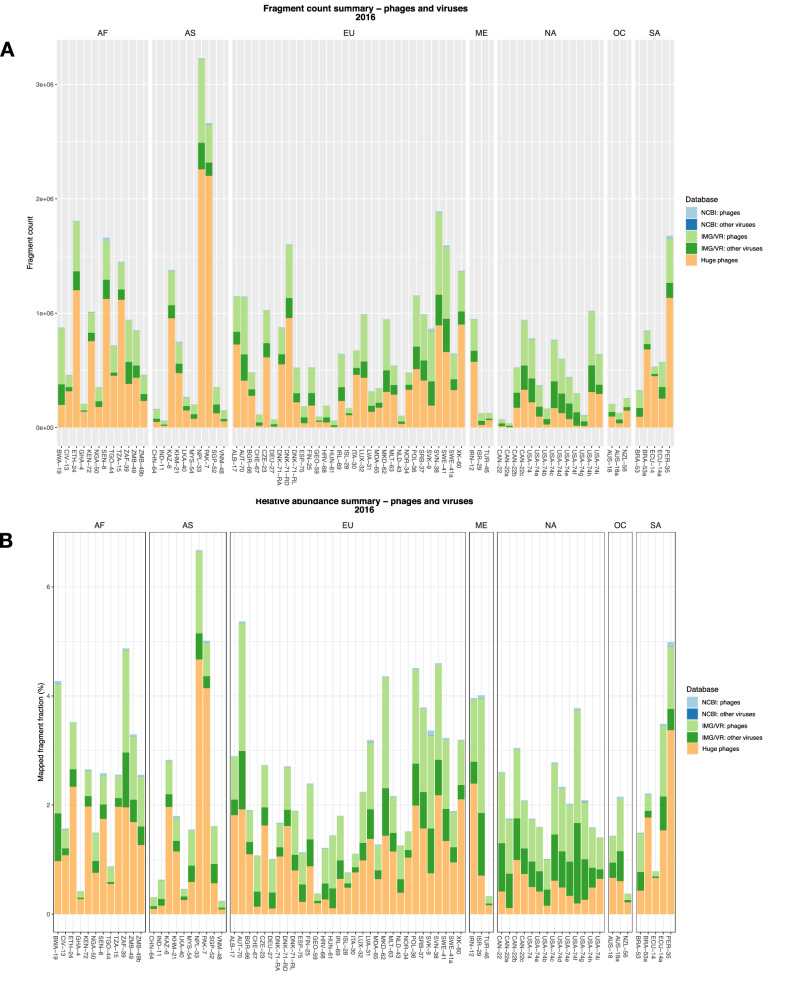
Fragment count (**A**) and relative abundance (**B**) summary of phages and viruses. *AF* Africa, *AS* Asia, *EU* Europe, *NA* North America, *ME* Middle East, *OC* Oceania, *SA* South America.

After mapping to bacteria and virus/phage databases, the vast majority of raw reads were mapped to the NCBI database of bacteria, followed by the IMG/VR database (Fig S5). Of the viral fraction, a larger proportion of reads were mapped to the huge phages database (Fig. 1A), followed by the IMG/VR database. Only very few reads were mapped to the NCBI virus database and mapping to the KVIT database was negligible. The latter was as expected, as the KVIT database includes only families of viruses infecting eukaryotic cells in addition to an unclassified branch.

The relative abundance profiles (Fig. 1B) for the various databases corresponded to those of fragment count summary, albeit the relative abundance of phages within the NCBI database was greater. The accumulated relative abundance of the IMG/VR database (incl. fragments) was in most cases comparable to that of the huge phages database.

### Regional distribution of phages

The number of samples contributing to each region is provided in Table S2. Most samples belonged to the European region, followed by the North American and African regions. From the NCBI database, phages constructed from African and Asia regions showed the highest matches to the NCBI database (approximately 70%). In contrast, with regards to the larger database of IMG/VR, Europe appeared to have the most phages identified with respect to both all phage-derived content (58.6%) and phage genomes (74.8%). Lastly, the number of phages identified from the huge phages database was similar in distribution to the NCBI database.

**Figure 2:**
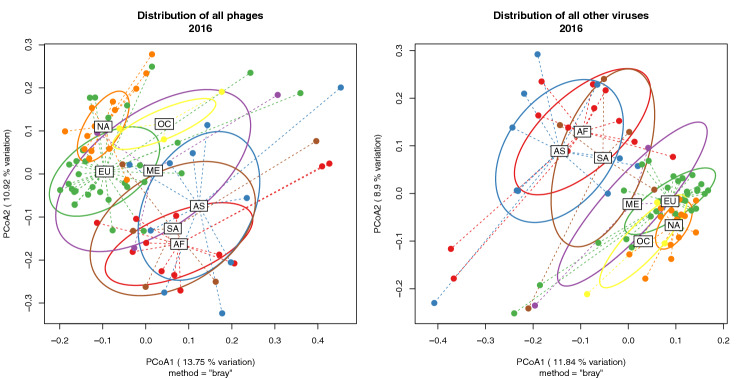
Regional distributions of phages (left) and other viruses (right) from all databases. *AF* Africa, *AS* Asia, *EU* Europe, *NA* North America, *ME* Middle East, *OC* Oceania, *SA* South America.

The regional distribution of all phages showed a clear regional separation; the same pattern was observed for all other viruses (Fig. 2). When considering the databases separately, the distribution of other viruses was dominated by phages from the IMG/VR database due to the significantly larger collection of viruses within this database (Fig S6). There was a clear regional separation of phages identified from the NCBI virus database, which was not observed with other viruses from the NCBI database (Fig S7).

### The effect of income on phage distribution

Datapoints driving the distribution in the three income classes were highly skewed towards high- and middle-income classes, as only 5 countries (6.4%) are classified as low-income according to the World Bank data of 2016 (Table S3), while most countries are classified as high-income (52.6%), or middle-income (41.0%).

Countries from Africa dominated the low-income class together with a single country from Asia (Nepal). Other Asian countries were within the middle-income class; Singapore was the only Asian country within the high-income class. All Middle Eastern and South American countries were within the middle-income class, and the European countries were predominantly within the high-income class. The North American and Oceania samples were all within the high-income class.

With regards to the total number of phages identified within each income class (Table 1), the middle-income class had the highest proportion of phages from the NCBI and huge phage databases, while the high-income class had the highest proportion of phages from the IMG/VR database. The low-income class exceeded the high-income class in the total number of phages only in the huge phages database.

**Table 1:** Proportion of phages within income classes.

	Total			100%			≥ 60%			Shared^a^		
	High	Middle	Low	High	Middle	Low	High	Middle	Low	High-middle	High-low	Middle-low
NCBI_count/total_ (%)	450/638 (70.5%)	565/638 (88.6%)	359/638 (56.2%)	45/638 (7.0%)	115/638 (18.0%)	14/638 (2.2%)	98/638 (15.3%)	207/638 (32.4%)	96/638 (15.0%)	113/638 (17.7%)	17/638 (2.7%)	162/638 (25.3%)
IMG/VR gen. _count/total_ (%)	3102/3699 (83.9%)	2979/3699 (80.5%)	1961/3699 (53.0%)	595/3699 (16.1%)	414/3699 (11.2%)	75/3699 (2.0%)	1186/3699 (32.1%)	645/3699 (17.4%)	429/3699 (11.6%)	957/3699 (25.9%)	170/3699 (4.6%)	337/3699 (9.1%)
Huge phages _count/total_ (%)	59/114 (51.8%)	99/114 (86.8%)	74/114 (64.9%)	4/114 (3.5%)	23/114 (20.2%)	6/114 (5.3%)	10/114 (8.8%)	26/114 (22.8%)	43/114 (37.7%)	12/114 (10.5%)	0/114 (0%)	21/114 (18.4%)

*gen.* genomes.

The total is the database total and not the income class total.

Number of shared phages are phages that in the excluded class contributes with ≤ 20% while the contribution for the included classes is ≥ 20%.

NCBI: 184, 67 and 269 not observed within the high-income, middle-income and low-income class, respectively.

IMG/VR genomes: 596, 719 and 1737 not observed within the high-income, middle-income and low-income class, respectively.

Huge phages: 55, 15 and 40 phages not observed within the high-income, middle-income and low-income class, respectively.

**Figure 3:**
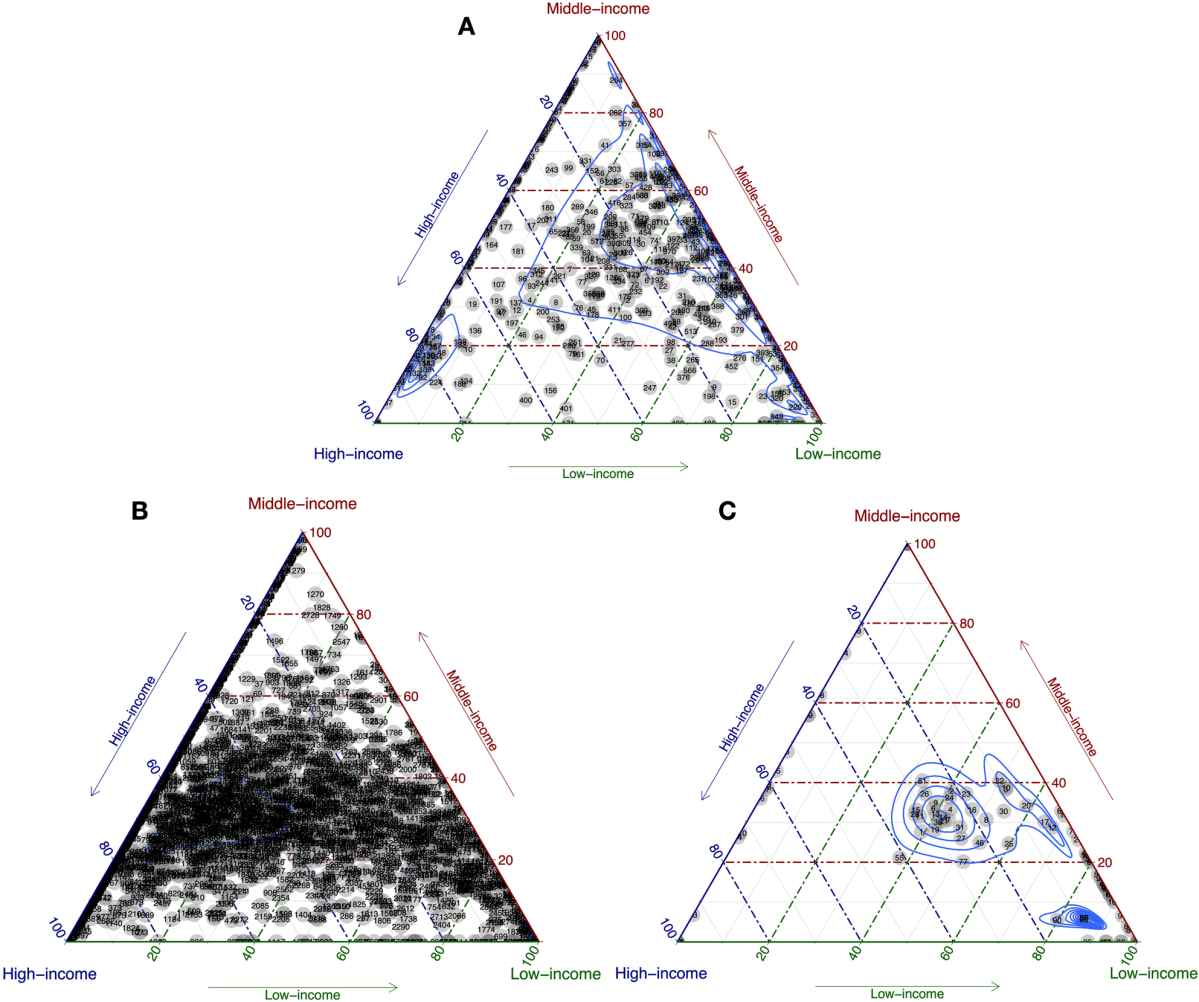
Distribution of NCBI phages (**A**), IMG/VR genomes (**B**) and huge phages (**C**) stratified by income class. Densities indicated by blue curves.

The distribution of phages identified from the NCBI virus database appeared to be skewed towards countries from middle- and low-income classes (Fig. 3A). With regards to the high-income class, 45 phages (7.0%) were observed only within this group, with 115 phages (18.0%) in the middle-income class and 14 phages (2.2%) in the low-income class (Table 1). The phages observed within the middle-income class were mainly *Salmonella* (17; 14.8%), *Pseudomonas* (14; 12.2%) and *Escherichia* (12; 11.3%), while the majority of phages observed within the high-income class were *Lactobacillus* (6; 13.3%) and *Mycobacterium* (5; 11.1%), and, in the low-income class, *Escherichia* (3; 21.4%) and *Lactobacillus* (3; 21.4%). For phages that distributed ≥ 60% to a given income class, the middle-income class had the largest number of phages followed evenly by that of high- and middle-income classes. The dense cluster distributed towards the high-income class contains a large number of phages infecting lactic acid fermenting bacteria.

With regards to phages identified from the larger database of IMG/VR (IMG/VR genomes), somewhat contrasting results were obtained with the majority of phages, contributing ≥ 60% to a given income class, observed within the high-income class, of which some were located within the dense cluster skewed towards this income class (Fig. 3B, faint blue density distribution).

Finally, the middle-income class had the highest number of unique phages identified from the huge phages database (Fig. 3C), most of which infect Proteobacteria (12; 52.2%), while the unique number of phages, infecting an assorted range of phyla, within the high- and low-income classes were only 4 (3.5%) and 6 (5.3%), respectively (Table 1).

### Phage abundance

**Figure 4:**
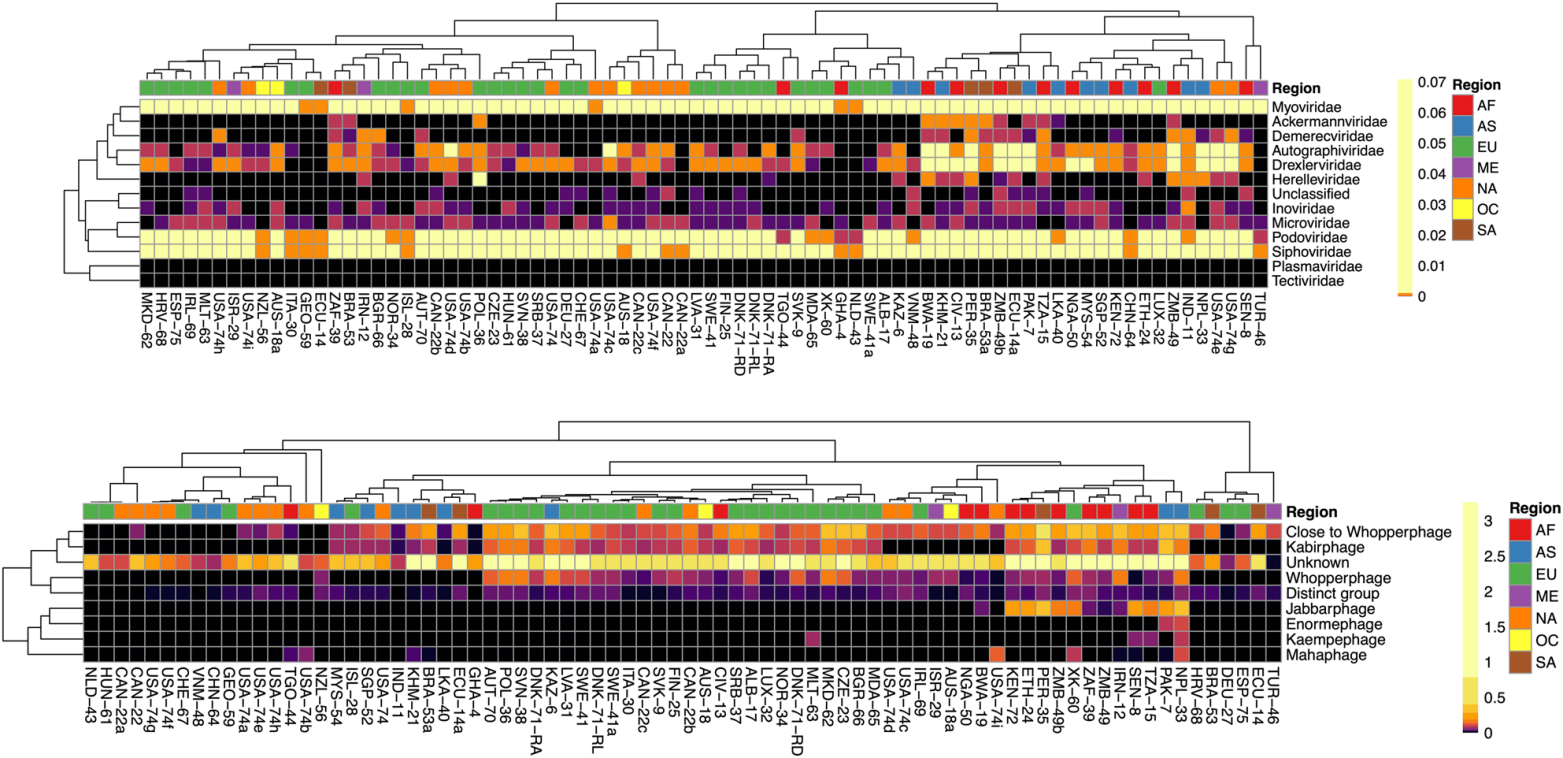
Pooled relative abundance of phages in their respective phage families (NCBI, top) and clades (huge phages, bottom). Intensities based on 10%- and 3.3%-quantiles, respectively. *AF* Africa, *AS* Asia, *EU* Europe, *NA* North America, *ME* Middle East, *OC* Oceania, *SA* South America.

The number of datapoints contributing to the pooled relative abundance of phages belonging to families of the NCBI virus database are provided in Table S4 and visualised in Fig. 4. Due to the poor annotation of the IMG/VR database, phages from this database could not be summarised according to family or clade.

Unsurprisingly, most datapoints were pooled for *Siphoviridae* (262 datapoints) and *Myoviridae* (173 datapoints), while *Podoviridae*, only represented by 66 datapoints, was succeeded by *Autographiviridae* (74 datapoints). Two single phages of low abundance belonged to *Plasmaviridae* and *Tectiviridae,* from two African and a North American sample, respectively. Moreover, the clustering of samples indicated a relatively strong correlation with regards to regionality, whereas Africa, Asia and South America tended to intersperse (Fig. 4, top).

An overview of the top 15 most abundant phages per sample from NCBI, IMG/VR genomes and the huge phages database is provided in Fig S8 to S10. Of the top 15 most abundant phages per sample from the NCBI database, the uncultured crAssphage (NC_024711, *Podoviridae*) and Salmonella phage SJ46 (NC_031129, *Myoviridae*) were common, and dominant, in almost all regions; India (IND-11) did not show a significant signal for the uncultured crAssphage (Fig S8). 13 seemingly associated Lactococcal phages and 3 Leuconostoc phages were highly clustered across some European countries, corresponding to some of those observed in the ternary plot of NCBI phages (Fig. 3A), including some surrounding Lactococcus and Leuconostoc phages. Some of these phages also appeared to be observed in some African countries, though with a lower abundance.

With regards to the custom clades of huge phages, only a low abundance of phages classified according to the terminase genes was observed, and most phages did not have a given classification (Table S4 and Fig. 4). The clade containing the largest phages within the dataset, i.e. Mahaphages, was present in low abundance.

### Phage phylogeny

**Figure 5:**
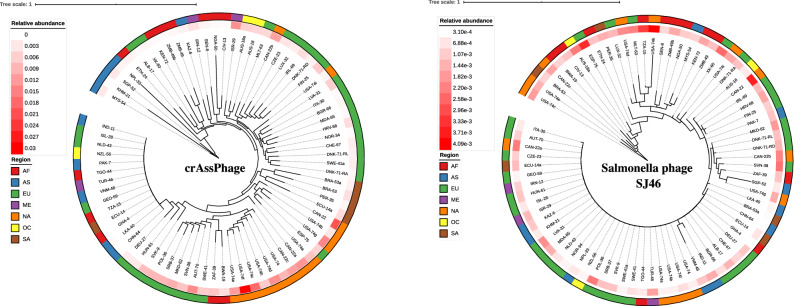
SNP trees of uncultured crAssphage (NC_024711, left) and Salmonella phage SJ46 (NC_031129, right). *AF* Africa, *AS* Asia, *EU* Europe, *NA* North America, *ME* Middle East, *OC* Oceania, *SA* South America.

It was only possible to generate SNP trees for the two most abundant phages from NCBI, i.e. the uncultured crAssphage (NC_024711) and Salmonella Phage SJ46 (NC_031129) (Fig. 5). The SNP trees were based on 2375 and 70 bases, respectively, and illustrate, that, at least for the uncultured crAssphage, there tends to be some regional clustering of phages sharing similar SNP profiles. For countries located within the tail of the phylogenetic tree, their relative abundance was too low to resolve differences (i.e. fragments covering the positions were most likely not observed), considering that the entire genome of the uncultured crAssphage (used as a reference) consists of approximately 97,000 bp. In comparison, the much fewer SNP positions identified within the Salmonella Phage SJ46, with a genome size of approximately 103,500 bp, exhibit only slight regional segregation, and many samples did not appear to contain the covered region, therefore a long trailing tail was observed from which SNPs could not be resolved. Furthermore, there was a tenfold difference in relative abundance between the two phages, the most abundant being from the NCBI database.

### Antimicrobial resistance gene abundance

For the ResFinder database (Fig S11, top), resistance gene conferring resistance towards compounds within the classes of tetracyclines, aminoglycosides, sulphonamides and phenicols was highly pronounced in the African, Asian, and South American regions, and to a lesser extent in Europe, North America, Oceania and Middle East. In contrast, macrolide, macrolide combination (Macro/Linco/StreptoB) and β-lactam resistance were more globally distributed, though with a slightly higher abundance in the former regions. With regards to the Functional Resistance database (Fig S11 bottom), the most common resistance classes were those conferring resistance towards the compounds of cefoxitin (cephalosporin; β-lactam), trimethoprim (antifolate), piperacillin (β-lactam), amoxicillin (β-lactam), and tetracycline (tetracycline), with penicillin (β-lactam), chloramphenicol (chloramphenicol) and co- trimoxazole (sulphonamide) and D. cycloserine (d-alanine analogue) being less abundant. No single region showed a greater degree of resistance to any one class, in contrast to the findings of ResFinder.

### Bacterial occurrence

The most abundant bacterial genera across most regions appeared to be *Acinetobacter, Acidovorax,* and *Cupriavidus* (Fig S12). A large cluster was apparent in the European and North American regions, which also largely clustered together, except for a few countries which interspersed with Africa, Asia and South America in smaller clusters. Neither the Middle East nor Oceania showed any degree of clustering.

The *Lactococcus* bacterial genus was, surprisingly, only present in low abundance in the countries where Lactococcal phages were represented (Fig S8), i.e. Slovakia (SVK-9), Sweden (SWE-41) and Latvia (LVA-31). The abundance of the *Lactococcus* genus was primarily confined to Europe and North America; it was observed globally to a lesser extent.

With regards to the Klebsiella and Escherichia phages observed within clusters within Africa, Asia and South America, there were no obvious differences in the abundance of their host genera, and both genera appeared to be found globally.

### Phage correlation to bacteria and ARGs

A significant correlation was observed for all viruses and bacteria; both in terms of correlation to each other (Table S5) and also with regards to ARGs. The highest correlation coefficients of approximately 0.90 (p < 0.001) were observed for the IMG/VR database, while correlation coefficient of more than 0.80 (p < 0.001) were observed within the NCBI database. The database of huge phages had correlation coefficients of approximately 0.70 (p < 0.001). Other viruses also exhibited significant correlations to both bacteria and ARGs.

Which potentially could be attributed to their low numbers in comparison to phages and their low abundance. Viral DNA is usually obtained using different procedures than those utilised for bacterial DNA purification.

## Discussion

It is neither uncommon nor surprising that only a small fraction of phages was observed. Due to the high diversity within phage nucleotide sequences and the fact that there is a certain overlap in phage and bacterial DNA, owing to the integration of prophages, adopting a targeted approach only allows for a very limited identification that may lead to false negatives with respect to phages. In addition, it is important to acknowledge the vast differences in database size. Comparison of the NCBI virus, NCBI bacteria and IMG/VR databases shows that only 2925 (as of June 2020) phage genomes have been assembled, while more than 370,000 bacterial genomes and more than 438,000 phage fragments have been assembled. Of the latter, 23,500 are high-quality draft or complete genomes. The differences in database sizes were evident, as many more fragments were mapped to the IMG/VR database in comparison to the NCBI database even when IMG/VR had competed against a much larger collection of sequences.

It has previously been established^17^ that employing an untargeted metagenomic approach will allow for a much wider identification of phages within metagenomic samples. This approach has its limitations, such as assembling an unbiased viral metagenome due to the vast diversity, while identifying the hosts of the assembled phages and performing annotations according to the ICTV classifications is not easily done. Consistent with this difficulty is the observation that the large majority of phages identified within this study only exhibited low coverages (Table S1). Other limitations are the inherent randomness of sequencing that is performed when undertaking a metagenomics approach, since bacterial DNA is far more abundant than that of phages^18^, and the fact that the DNA purification protocol must be adapted to the given phage environment, which is not the case for the data employed in this study. In addition, phages are usually purified from the viral portion of the sample. Hence, purification and identification of phages using a metagenomics approach require carefully considered protocols and pipelines bearing in mind the aim of the study. Thus, phages expected to be observed in the present study are likely those associated with bacteria. However, the specific phage lifecycle may be dictated by external factors such as temperature and chemicals such as antimicrobial residues, both of which influence municipal wastewater. The global sewage study from which these data originate^11^ detected antimicrobial residues. A previous study has found that concentrations of ciprofloxacin, for example, in wastewater may be high enough to induce the SOS response in bacteria, which in turn affects the activation of prophages^19^. It is therefore likely that many phages can be regarded as free-living.

Despite the limitations mentioned above the results show that a country’s income (GNI per capita) could play a role in the distribution of bacteriophages. The phage composition of the middle-income class was distributed somewhat between the two other classes, but generally shared more phages with the low-income class. Both classes had Escherichia phages among their unique phages. The low-income class only appeared to share a small number of phages with the high-income class, although both classes had Lactobacillus phages (another lactic acid fermenting bacterial genus) among their unique phages. A possible explanation for this observation could be differences in dietary intake, a parameter not investigated in the present project, but that is shown to be—at least locally—affected by income^20^ and culture, which in turn could affect the intestinal microbiota, and thus potentially phages. This hypothesis is supported by the observation of a higher prevalence of phages infecting lactic acid fermenting bacteria, such as Lactococcus and Leuconostoc phages, in high-income countries and certain regions associated with these which are known to consume larger amounts of dairy products than countries in other income classes^21^. Furthermore, antibiotics are usually consumed in larger amounts within high-income countries compared to middle- and low-income countries^22^, in which insufficient use, i.e. not covering a full course of treatment, both can lead to resistance. According to the World Health Organisation (WHO), amoxicillin and amoxicillin/clavulanic acid are the most frequently used antibiotics globally, consistent with the finding that the abundance of amoxicillin resistance genes detected within the samples were among the highest. There is an increase in resistance towards these specific antibiotics in some *Lactococcus* species^23^, and there is increasing evidence that foodborne lactic acid species are overlooked with regards to the dissemination of ARGs^24^. Some broad-spectrum antibiotics are classified by the WHO as Watch antibiotics, due to their high potential in causing antibiotic resistance phenotypes. It is unfortunate, therefore that cefoxitin (cephalosporin), a Watch antibiotic, was one of the most abundant resistance phenotypes observed in the Functional Resistance database. Most *Lactococcus* sp. are intrinsically resistant towards cefoxitin. There are also other confounding factors such as different climates that are associated with countries and could directly or indirectly affect the decay rates of microorganisms in sewage.

Unfortunately, only SNP trees could be resolved for the uncultured crAssphage and the Salmonella phage SJ46. It is not surprising that the crAssphage appeared to be the most abundant, as it reportedly constitutes a class of highly abundant phages infecting the genus *Bacteroides*^25^, the most abundant bacterial genera in the human gut microbiota^26^. The identification of the Salmonella phage SJ46 is somewhat surprising, however, as the *Salmonella* genus is not among the most abundant bacterial genera within the dataset. The occurrence of Salmonella phage SJ46 is likely explained by its relatedness to the E. coli P1 phage^27^; a temperate phage that does not integrate into the host genome, but merely resides as a plasmid. As some phages have a broader host range, Salmonella phage SJ46 could potentially also target *E. coli* strains, which are among the most abundant bacteria in sewage. Many phages observed within the present dataset appeared to be Escherichia phages.

Whether there is truly a correlation between phages and the dissemination of ARGs in wastewater is still unknown. Unfortunately, due to the low abundance of phages identified within this study, definite conclusions cannot be made. The observation of a significant correlation between phages and other viruses, which should not correlate to either bacteria or ARGs, could arise from the fact that other viruses tend to be observed only in very low numbers compared to phages, implying a false positive (type 1 error). However, it has previously been suggested that there is a negative selection for resistance genes, as phages tend to select for genes acting in carbon flux pathways for their own benefit in reproduction^9^. This has mainly been investigated for oceanic surface and deep water phages, in which bacterial nutrition is limited, which is not the case for wastewater influent. However, a positive selection for genes acting in carbon flux pathways could suggest a possibility that phages may not be significantly correlated to ARGs. Nevertheless, the distribution of Lactococcal phages in the present study in relation to the increasing resistance in *Lactococcus* species tend to indicate that there could be a significant correlation between phages and ARGs, especially considering the increasing resistance phenotypes observed in these bacteria.

## Methods

### Urban sewage samples

A total of 81 samples from global wastewater surveillance was downloaded from the European Nucleotide Archive (ENA) with study number PRJEB13831, and sample accession numbers SAMEA4527599–SAMEA4527679^11^. The samples contained raw sequencing reads from influent wastewater in the period from January to December 2016 (Table S6). The DNA of the samples had been fragmented and sequenced on an Illumina HiSeq 3000 NGS platform and contained paired end reads. The samples were trimmed using BBDuk2, hosted and maintained by JGI^28^ to a minimum quality score (Phred64) of 20 and read length of 50 base pairs. Singletons, i.e. reads that are not paired, were sorted into a separate file for each sample.

### Bacteriophage database selection

For the present study reference sequences from some of the largest databases containing viral genetic sequences or fragments constitute those of RefSeq and IMG/VR hosted by NCBI and JGI, respectively, were used. The RefSeq database contains approximately 9700 viral genomes of which phage genomes constitute approximately 3000 as of June 2020. The composition of phage families in the NCBI database is provided in Table S7. The IMG/VR database contains approximately 760,000 viral sequences and fragments from various bio-projects, including sequences from NCBI, of which approximately 438,000 are of phage origin. Of these, 23,500 are of high-quality, more than 90% completed draft, or entirely completed genomes. The RefSeq database is an open access, annotated, curated and non-redundant database, while the IMG/VR database is an open access database but is less curated and is not annotated with regards to host-virus interactions and taxonomy. In addition, the KVIT database, comprising sequences from RefSeq and GenBank, was included in the comparative analysis of non-phage viruses, as it only contains viral families infecting eukaryotic cells. Additionally, a custom database containing huge phages across Earth’s ecosystem^2^ was used. The database contained 351 genomes of huge phages with genome sizes ranging from 200 to 735 kb.

### Read mapping

Trimmed raw reads were mapped to the different databases including bacteria and virus/phage databases using K-mer mapping (KMA)^29^, as KMA is optimised for mapping against redundant databases. With regards to phages, it was expected that at least some overlap between bacteria and phages would be observed, due to the fact that a substantial portion of the bacterial genome is believed to be of phage origin^30^. In comparison to other commonly used mapping tools, such as Bowtie and BWA-MEM^31^, KMA appears superior both in accuracy, but also in speed, in addition to being comparable in terms of memory consumption.

Prior to read mapping, all reference sequences in FASTA format were indexed in KMA with an indexing k-mer size of 16. The size 16 k-mer is a proper k-mer length as it will maintain selectivity as well as specificity.

### Regional distribution of phages

Fragment count and relative abundance from read mapping against viruses and phages in RefSeq, IMG/VR, and the customised database of huge phages were visualised in stacked bar plots using the *ggplot* package in R. The number of phages contributing to each region was found by summing the relative abundance of a given phage across samples belonging to a given region. Phages having a sum greater than 0 were regarded as having been observed within that region. Their percentages of the total number identified for a given database were subsequently calculated.

The regional distribution of phages was visualised through a principal coordinate analysis (PCoA) of the relative abundance of phages, viruses and bacteria. PCoA was chosen over that of e.g. PCA due to the low abundance and many null values observed, making the Bray–Curtis dissimilarity more suitable for ordination, and in turn making PCoA more suitable for viewing dissimilarities. The PCoA plots were generated on Hellinger standardised relative abundance to account for differences in abundance, and the dissimilarities between samples were, as mentioned previously, computed using Bray–Curtis dissimilarities. The *betadisper* function of the *vegan* package in R was used to reduce the original non-Euclidean distances (or more appropriately dissimilarities) of the Bray–Curtis dissimilarity matrix to principal coordinates, and the variance contributions of each principal coordinate were calculated using the eigenvals function of the *vegan* package.

### Distribution of phages by income country

Countries were classified according to the World Bank classification of low, middle and high income^32^ in the year 2016 to match the dataset. The middle-income classification includes sub-classifications of lower-middle-income and upper-middle-income classes, which were collectively classified as middle-income. Datasets annotated by income class were analysed in R whereby datapoints were averaged to each categorical income class. Data were subsequently visualised in the *ggplot2* extension package *ggtern* to create ternary plots of individual phage distributions. The phage distributions were found using the following formula: (mean relative abundance of a phage in an income class/ sum of mean relative abundance for a phage across all income classes)*100. The densities of the distributions were calculated using an inherent function of *ggtern*.

### Phage abundance

The relative abundance of phages was visualised for the top 15 most abundant phages per sample and pooled for all phages in each sample within each family (for NCBI phages) or clade (for huge phages) using the R package *pheatmap*. Due to the poor annotation of IMG/VR no data pooling was performed for this database. The very low abundance of phages made log-transformation insignificant, thus raw values were used for visualisation. Instead, colour intensities within the heatmaps were generated based on abundance quantiles (Table S8) to show the distribution of the phages. Quantiles were determined based on the visual representation, with the smallest possible margins (percentages). The samples were clustered according to Bray–Curtis dissimilarities of Hellinger standardised relative abundance, while phages were clustered according to their Pearson correlation, to explore how phage abundance was associated.

### Phage phylogeny

Only the most abundant phages were used for further phylogenetic analysis. The analysis was conducted using CSIPhylogeny^33^ and phyML^34^, and visualised using iTOL^35^. CSIPhylogeny was used to perform variant calling and subsequent sequence alignment by using the most abundant phages as references and sewage metagenomics raw reads as input. The tree topology structure was generated using PhyML on basis of the HKY85 substitution model and SPR tree topology heuristic with 100 bootstrap replicates. An attempt to find the most optimal nucleotide substitution model was done using jmodeltest^36^, but did not succeed, therefore the default substitution model was selected.

### Antimicrobial resistance gene distribution and abundance

Metagenomic reads were mapped against the ARG database ResFinder^37^. The regional distribution of ARGs was visualised in a PCoA of Bray–Curtis dissimilarities, based on the relative abundance in Fragments Per Kilobase of gene per Million mapped reads (FPKM) that had been Hellinger transformed using the *vegan* package. In addition, FPKM of resistance genes were pooled for each sample by resistance classes and a heatmap produced by log-transformed relative abundance that had been given a pseudo-count prior to log-transformation. Column clustering was performed on a Bray–Curtis dissimilarity matrix of Hellinger transformed FPKM values to account for low abundance genes, while row clustering was performed using Pearson correlation.

### Bacterial occurrence

The top 15 most abundant bacterial genera per sample were visualised in R using the package *pheatmap* of the log-transformed bacterial abundance. Columns were clustered according to Bray–Curtis dissimilarities of Hellinger standardised relative abundance, while rows were clustered according to their Pearson correlations. Colour intensities were generated similar to those of phages, by quantiles (Table S9) in effect rendering the log-transformation obsolete as using either raw or log-transformed data would produce the same output based on the distribution.

### Procrustes correlation analysis

The Procrustes correlation analysis was performed using R package *mganalysis*^38^. No visualisation was made subsequent to analysis and only correlation values and significance levels on a 95% confidence interval were calculated. The analysis was performed on data of relative abundance for phages correlated to the relative abundance of bacteria or the FPKM of ARGs.

## Supplementary information

Supplementary Information.
