# Supplementary material for: Metagenomics analysis of bacteriophages and antimicrobial resistance from global urban sewage
Source: Sci Rep. 2021 Jan 15;11:1600. doi: 10.1038/s41598-021-80990-6 (PMC7810828; doi:10.1038/s41598-021-80990-6)
Supplement: Supplementary file 1 — Supplementary Information. [file 41598_2021_80990_MOESM1_ESM.pdf]

Metagenomics analysis of bacteriophages and antimicrobial resistance from global urban sewage

Josephine E.S. Strange[1], Pimlapas Leekitcharoenphon[1*], Frederik Duus Møller[1], Frank M. Aarestrup[1]

[1]National Food Institute, Technical University of Denmark, WHO Collaborating Center for Antimicrobial Resistance in Food borne Pathogens and Genomics, European Union Reference Laboratory for Antimicrobial Resistance, Kgs. Lyngby, Denmark.

Supplementary Table 1

Coverage and depth of coverage distributions

		NCBI phages	IMG/VR genome	Huge phages
Coverage (%)	Mean (SD)	1.21 (4.96)	2.5 (7.78)	2.09 (8.09)
	Min;Max	0;96.08	0;97.62	0;97.25
Depth of coverage	Mean (SD)	0.03 (0.34)	0.07 (0.72)	0.18 (2.83)
	Min;Max	0;33.28	0;190.27[1]	0;176.58[2]

[1]The phage from IMG/VR genome having a depth of 190.27 is X3300014750_____Ga0134554_100011 with a sequence length of 103,348 bp. The phage was found in various datasets with coverages ranging between 3.35% to 93.55%

[2]The phage from custom huge phages having a depth of 176.58 is F27_PHAGE_CIR_34_39 with a sequence length of 200,395 bp. The phage was found in various datasets with coverages ranging between 0% to 59.14%.

Supplementary Table 2

Number of samples and phages within each region

	Total	AF	AS	EU	ME	NA	OC	SA
**Number of samples**	78	12	10	31	3	14	3	5
				**Number of phages**				
**NCBI**	638	447 (70.1%)	454 (71.2%)	373 (58.5%)	178 (27.9%)	313 (49.1%)	129 (20.2%)	382 (59.9%)
**IMG/VR all**	54893	21392 (39.0%)	19794 (36.1%)	32157 (58.6%)	10461 (19.1%)	26354 (48.0%)	8847 (16.1%)	15834 (28.8%)
**IMG/VR genomes**	3699	2287 (61.8%)	2182 (59.0%)	2767 (74.8%)	1281 (34.6%)	2436 (65.9%)	993 (26.8%)	1881 (50.9%)
**Huge phages**	114	60 (52.6%)	81 (71.1%)	64 (56.1%)	32 (28.1%)	31 (27.2%)	13 (11.4%)	56 (49.1%)

AF, Africa; AS, Asia; EU, Europe; NA, North America; ME, Middle East, OC, Oceania; SA South America

Supplementary Table 3

Distribution of countries within income classes

Income class	AF	AS	EU	NA	ME	OC	SA	Total
High	0	1	23	14	0	3	0	41 (52.6%)
Middle	8	8	8	0	3	0	5	32 (41.0%)
Low	4	1	0	0	0	0	0	5 (6.4%)

For classification of countries please refer to Supplementary Table 1. Percentages are given as the percent of total samples.
AF, Africa; AS, Asia; EU, Europe; NA, North America; ME, Middle East; OC, Oceania; SA, South America

Supplementary Table 4

Summary of pooled datapoints within phage families and clades

Database	Order/Clade	Family	Number of datapoints (phages)
NCBI virus	*Caudovirales*	*Siphoviridae*	262
	Caudovirales	*Myoviridae*	173
	Caudovirales	*Autographiviridae*	74
	Caudovirales	*Podoviridae*	66
	Caudovirales	*Drexlerviridae*	17
	Tubulavirales	*Inoviridae*	11
	Petitvirales	*Microviridae*	10
	Caudovirales	*Demerecviridae*	10
	Caudovirales	*Herelleviridae*	6
	Caudovirales	*Ackermannviridae*	5
	Unclassified	Unclassified†	2
	Unclassified	*Plasmaviridae*	1
	Kalamavirales	*Tectiviridae*	1
Custom huge phages	Unknown		80
	Mahaphage		9
	Kabirphage		6
	Close to Whopperphage		6
	Whopperphage		5
	Jabbarphage		4
	Kaempephage		2
	Distinct group		2
	Enormephage		1

†The two unclassified phages were that of Enterobacteria phage P4 (NC_001609) and Hydrogenobaculum phage strain HP1 (NC_028651)

Supplementary Table 5

Procrustes correlation

	Correlation coefficient	p-value
NCBI phages & bacteria	0.8148	0.001
IMG/VR phages & bacteria	0.8956	0.001
Custom huge phages & bacteria	0.7054	0.001
Bacteria & ARGs	0.8813	0.001
NCBI phages & ARGs	0.8488	0.001
IMG/VR phages & ARGs	0.8956	0.001
Custom huge phages & ARGs	0.7379	0.001
NCBI other viruses & bacteria	0.7167	0.001
NCBI other viruses & ARGs	0.7619	0.001
IMG/VR other viruses & bacteria	0.8896	0.001
IMG/VR other viruses & ARGs	0.9033	0.001

Supplementary Table 6

Overview of sample annotations and temperatures at day of sample collection

Sample	Sample accession	Country	City	Region	Income class	Collection date	Temperature (°C)		
							Sample	Transport	Storage
ALB-17	SAMEA4527599	Albania	Tirana	EU	Upper middle	02 January	12	12	-73
AUS-18	SAMEA4527600	Australia	Woden	OC	High	02 April	19.3	4	-80
AUS-18a	SAMEA4527601	Australia	Melbourne	OC	High	02 August	23.8	4	-80
AUT-70	SAMEA4527602	Austria	Vienna	EU	High	16 February	7	18	-80
BGR-66	SAMEA4527603	Bulgaria	Sofia	EU	Upper middle	15 February	5	4	-70
BRA-53	SAMEA4527604	Brazil	Belém	SA	Upper middle	16 February	NR	4	-70
BRA-53a	SAMEA4527605	Brazil	Belém	SA	Upper middle	15 February	28	4	-80
BWA-19	SAMEA4527606	Botswana	Palapye	AF	Upper middle	25 January	32	4	-80
CAN-22	SAMEA4527607	Canada	Regina	NA	High	26 January	-5.6	4	-80
CAN-22a	SAMEA4527608	Canada	Calgary	NA	High	02 January	-4	4	-80
CAN-22b	SAMEA4527609	Canada	Toronto	NA	High	02 January	4	4	-80
CAN-22c	SAMEA4527610	Canada	Ottawa	NA	High	26 January	-0.7	4	-80
CHE-67	SAMEA4527611	Switzerland	Liebefeld	EU	High	27 January	3.8	-20	-70
CHN-64	SAMEA4527612	China	Guangzhou	AS	Upper middle	28 January	13	13	-70
CIV-13	SAMEA4527613	Côte d’Ivoire	Abidjan	AF	Lower middle	25 January	NR	5	-80
CZE-23	SAMEA4527615	Czechia	Brno	EU	High	02 January	11	9	-75
DEU-27	SAMEA4527616	Germany	Berlin	EU	High	02 August	NR	NR	-80
DNK-71-RA	SAMEA4527617	Denmark	Avedøre	EU	High	03 February	4	-20	10
DNK-71-RD	SAMEA4527618	Denmark	Damhusåen	EU	High	03 February	4	-20	10
DNK-71-RL	SAMEA4527619	Denmark	Lynetten	EU	High	03 February	4	-20	10
ECU-14	SAMEA4527620	Ecuador	Quito	SA	Upper middle	02 January	24	15	-80
ECU-14a	SAMEA4527621	Ecuador	Galápagos	SA	Upper middle	02 June	28	28	-80
ESP-75	SAMEA4527622	Spain	Barcelona	EU	High	02 October	14.5	10	-75
ETH-24	SAMEA4527623	Ethiopia	Addis Ababa	AF	Low	02 January	27	4	-80
FIN-25	SAMEA4527624	Finland	Helsinki	EU	High	26 January	-1	6	-80
GEO-59	SAMEA4527625	Georgia	Tbilisi	EU	Lower middle	25 January	1	4	-80

Sample	Sample accession	Country	City	Region	Income class	Collection date	Temperature (°C)		
							Sample	Transport	Storage
GHA-4	SAMEA4527626	Ghana	Tamale	AF	Lower middle	30 January	30	4	-20
HRV-68	SAMEA4527628	Croatia	Zagreb	EU	Upper Middle	02 January	14	42	-82
HUN-61	SAMEA4527629	Hungary	Budapest	EU	High	02 February	11	10	-80
IND-11	SAMEA4527630	India	Cochin	AS	Lower middle	27 January	32	15	-80
IRL-69	SAMEA4527631	Ireland	Dublin	EU	High	02 October	8	4	-80
IRN-12	SAMEA4527632	Iran	Tehran	ME	Upper middle	02 February	11	4	-80
ISL-28	SAMEA4527633	Iceland	Reykjavik	EU	High	02 February	-3	4	-77
ISR-29	SAMEA4527634	Israel	Jerusalem	ME	High	31 January	3	4	-80
ITA-30	SAMEA4527635	Italy	Rome	EU	High	02 February	13	4	-80
KAZ-6	SAMEA4527636	Kazakhstan	Almaty	AS	Upper middle	02 February	5	10	-80
KEN-72	SAMEA4527637	Kenya	Thika	AF	Lower middle	02 August	25	10	-80
KHM-21	SAMEA4527638	Cambodia	Phnom Penh	AS	Lower middle	02 May	34	34	-80
LKA-40	SAMEA4527639	Sri Lanka	Colombo	AS	Lower middle	28 February	31	30	-80
LUX-32	SAMEA4527640	Luxembourg	Belvaux	EU	High	02 January	9	6	-80
LVA-31	SAMEA4527641	Latvia	Riga	EU	High	02 January	3	4	-80
MDA-65	SAMEA4527642	Moldova	Chișinău	EU	Lower middle	04 February	NR	NR	NR
MKD-62	SAMEA4527643	Macedonia	Skopje	EU	Upper middle	02 February	19	4	-80
MLT-63	SAMEA4527644	Malta	Saint Venera	EU	High	02 January	17	10	-80
MYS-54	SAMEA4527645	Malaysia	Kuala Lumpur	AS	Upper middle	02 April	29	4	-80
NGA-50	SAMEA4527646	Nigeria	Lagos	AF	Lower middle	02 February	NR	4	-80
NLD-43	SAMEA4527647	Netherlands	Bilthoven	EU	High	27 January	11	5	-50
NOR-34	SAMEA4527648	Norway	Oslo	EU	High	02 March	1	4	-70
NPL-33	SAMEA4527649	Nepal	Kathmandu	AS	Low	30 January	26	26	-80
NZL-56	SAMEA4527650	New Zealand	Dunedin	OC	High	27 January	14	4	-80
PAK-7	SAMEA4527652	Pakistan	Karachi	AS	Lower middle	27 January	24	25	-80
PER-35	SAMEA4527653	Peru	Lima	SA	Upper middle	02 April	29	4	-80
POL-36	SAMEA4527651	Poland	Puławy	EU	High	02 February	8	4	-80
SEN-8	SAMEA4527654	Senegal	Dakar	AF	Low	02 February	NR	7	-80
SGP-52	SAMEA4527655	Singapore	Singapore	AS	High	02 March	31	30	-80
SRB-37	SAMEA4527656	Serbia	Belgrade	EU	Upper middle	27 January	10	4	-80
SVK-9	SAMEA4527657	Slovakia	Bratislava	EU	High	02 February	10	10	-80
SVN-38	SAMEA4527658	Slovenia	Ljubljana	EU	High	25 January	0	6	-70

Sample	Sample accession	Country	City	Region	Income class	Collection date	Temperature (°C)		
							Sample	Transport	Storage
SWE-41	SAMEA4527659	Sweden	Uppsala	EU	High	02 February	5	5	-75
SWE-41a	SAMEA4527660	Sweden	Gothenburg	EU	High	02 November	3	15	-76
TGO-44	SAMEA4527662	Togo	Lomé	AF	Low	26 January	30	30	-80
TUR-46	SAMEA4527663	Turkey	Ankara	ME	Upper middle	29 January	12	6.1	-83
TZA-15	SAMEA4527664	Tanzania	Moshi	AF	Low	02 March	27	23	-80
USA-74	SAMEA4527665	USA, Georgia	Atlanta	NA	High	02 February	24.4	4	-25
USA-74a	SAMEA4527666	USA, Washington	Seattle	NA	High	02 January	7.8	4	-80
USA-74b	SAMEA4527667	USA, Illinois	Chicago	NA	High	24 February	3.9	5	-80
USA-74c	SAMEA4527668	USA, Texas	El Paso	NA	High	23 February	16.1	4	6
USA-74d	SAMEA4527669	USA, Oregon	Portland	NA	High	02 October	15.5	NR	4
USA-74e	SAMEA4527670	USA, Texas	El Paso	NA	High	22 February	16.1	4	6
USA-74f	SAMEA4527671	USA, Texas	El Paso	NA	High	22 February	16.1	4	6
USA-74g	SAMEA4527672	USA, Texas	El Paso	NA	High	23 February	16.1	4	6
USA-74h	SAMEA4527673	USA, Colorado	Adams County	NA	High	NR	NR	NR	NR
USA-74i	SAMEA4527674	USA, Colorado	Boulder	NA	High	28 March	13.9	4	4
VNM-48	SAMEA4527675	Vietnam	Ho Chi Minh	AS	Lower middle	27 January	NR	NR	NR
XK-60	SAMEA4527676	Kosovo	Pristina	EU	Lower middle	02 March	11	6	-80
ZAF-39	SAMEA4527677	South Africa	Johannesburg	AF	Upper middle	02 April	30	4	-70
ZMB-49	SAMEA4527678	Zambia	Lusaka	AF	Lower middle	25 January	26.7	4	4
ZMB-49b	SAMEA4527679	Zambia	Kitwe	AF	Lower middle	01 December	27	4	4

Sample: Temperature of sample at time of sampling.

NR: Not reported; AF, Africa; AS, Asia; EU, Europe; ME, Middle East; NA, North America; OC, Oceania; SA, South America.

Supplementary Table 7

Phage family composition within the NCBI database†

Order	Family	Number of phage members
**Caudovirales**	*Ackermannviridae*	23
	Autographiviridae	349
	Chaseviridae	7
	Demerecviridae	55
	Drexlerviridae	71
	Myoviridae	623
	Podoviridae	241
	Siphoviridae	1410
	Herelleviridae	120
	Unclassified	2
**Kalamavirales**	*Tectiviridae*	7
**Microvirales**	*Microviridae*	61
**Tubulavirales**	*Inoviridae*	43
**Vinavirales**	*Corticoviridae*	2
**Unclassified**	*Plasmaviridae*	1

†Numbers as of June 2020. As the NCBI database utilised for mapping within the study is from March 2019, the numbers are likely less than the ones reported here; it was not possible to obtain a data on the database composition.

Supplementary Table 8

Quantile breaks and their corresponding percentages

Database	Quantile breaks	Colour representation[†]
NCBI phages	200	0.5%
IMG/VR genomes	15	6.7%
Huge phages	30	3.3%
NCBI families	10	10%
Clades of huge phages	30	3.3%

[†]Percentage of datapoints in the distribution represented by a single colour, e.g. the top 0.5% of the datapoints for NCBI phages are represented by pale yellow.

Supplementary Table 9

Quantile breaks of NCBI bacterial genera

Database	Quantile breaks	Colour representation[†]
NCBI bacterial genera	20	5%

[†]Percentage of datapoints in the distribution represented by a single colour, e.g. top 5% of datapoints are represented by pale yellow.

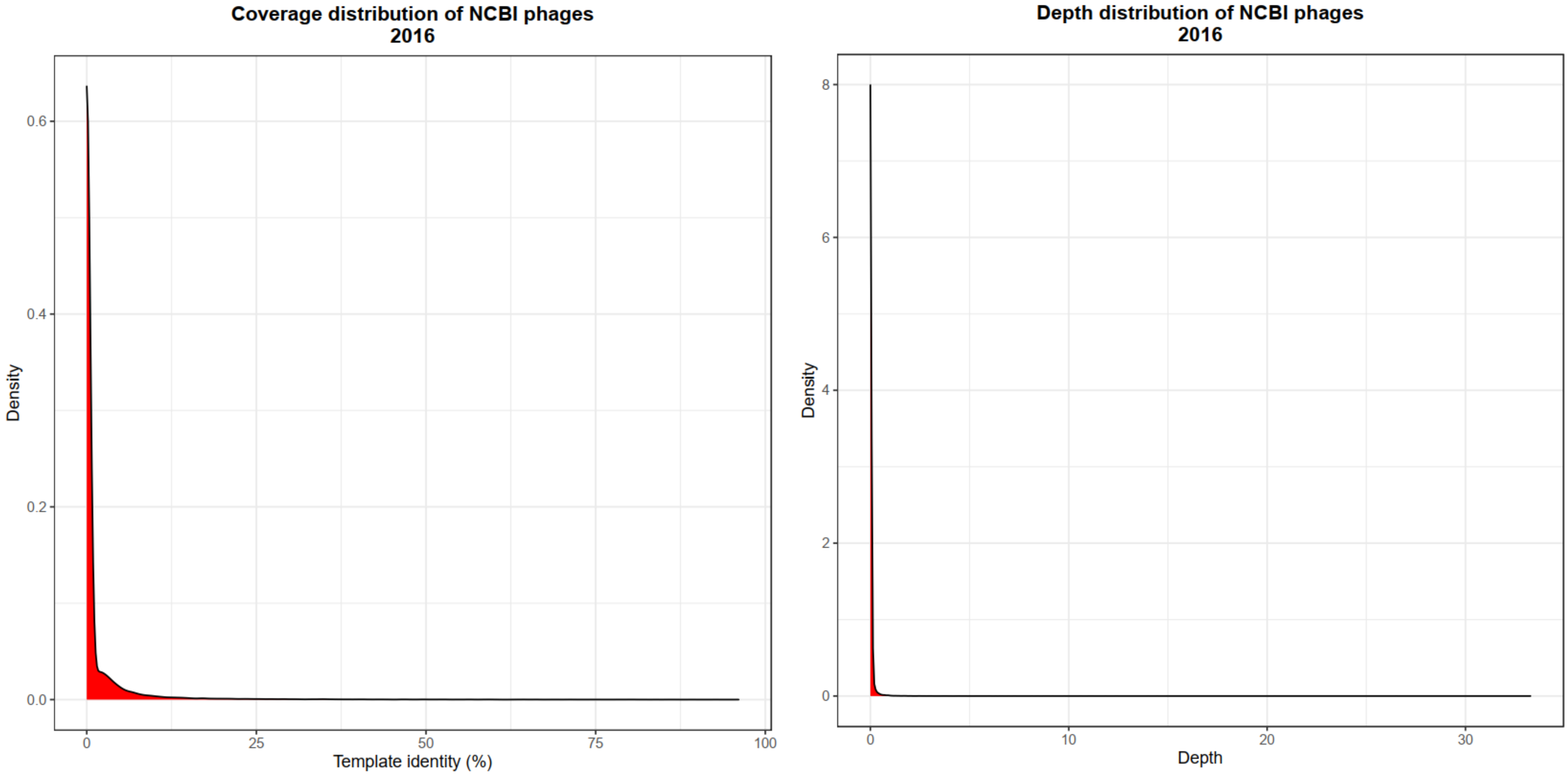

Supplementary Figure 1 Coverage (left) and depth (right) distributions for NCBI phages

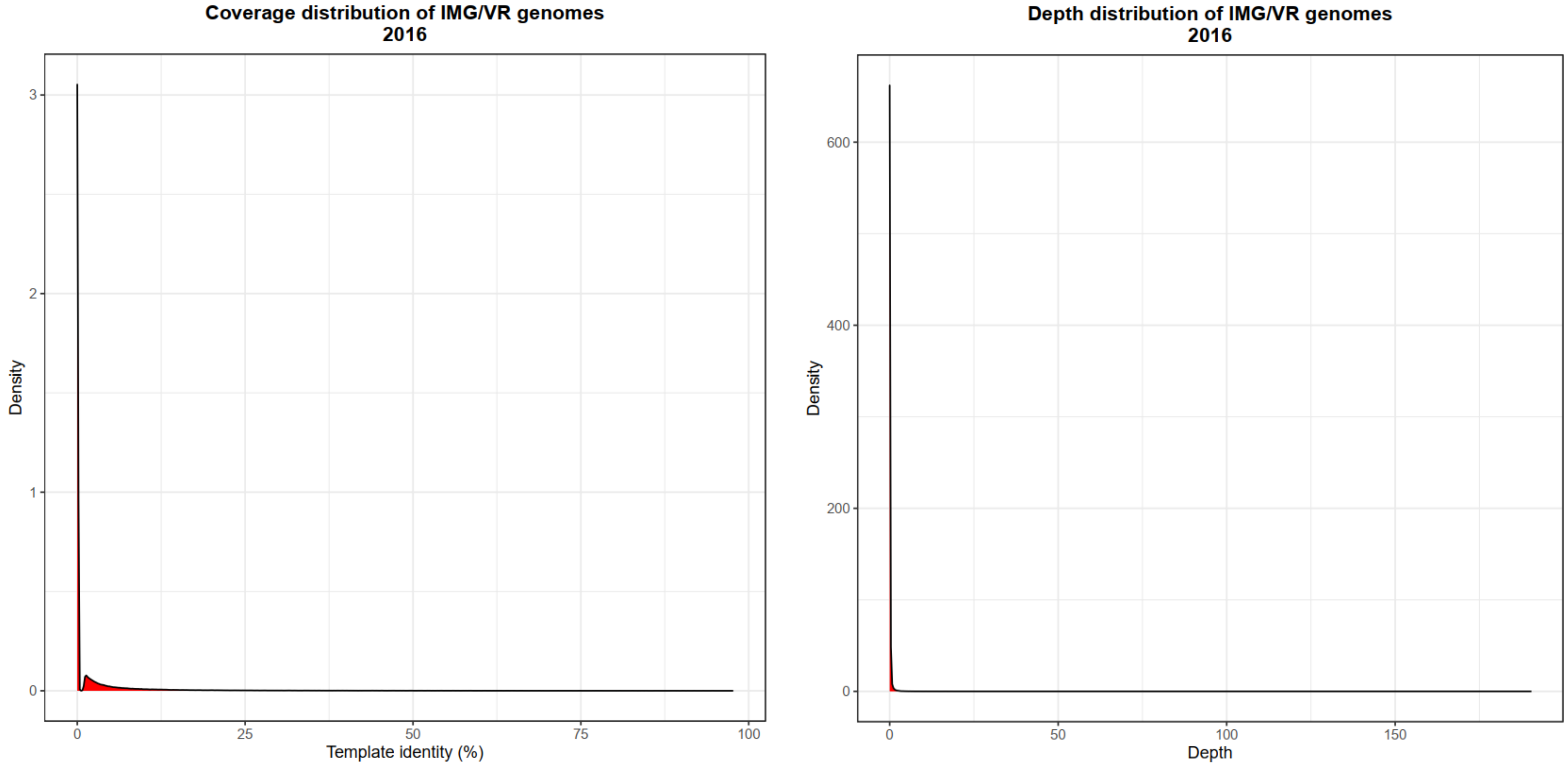

Supplementary Figure 2 Coverage (left) and depth (right) distributions for IMG/VR genomes

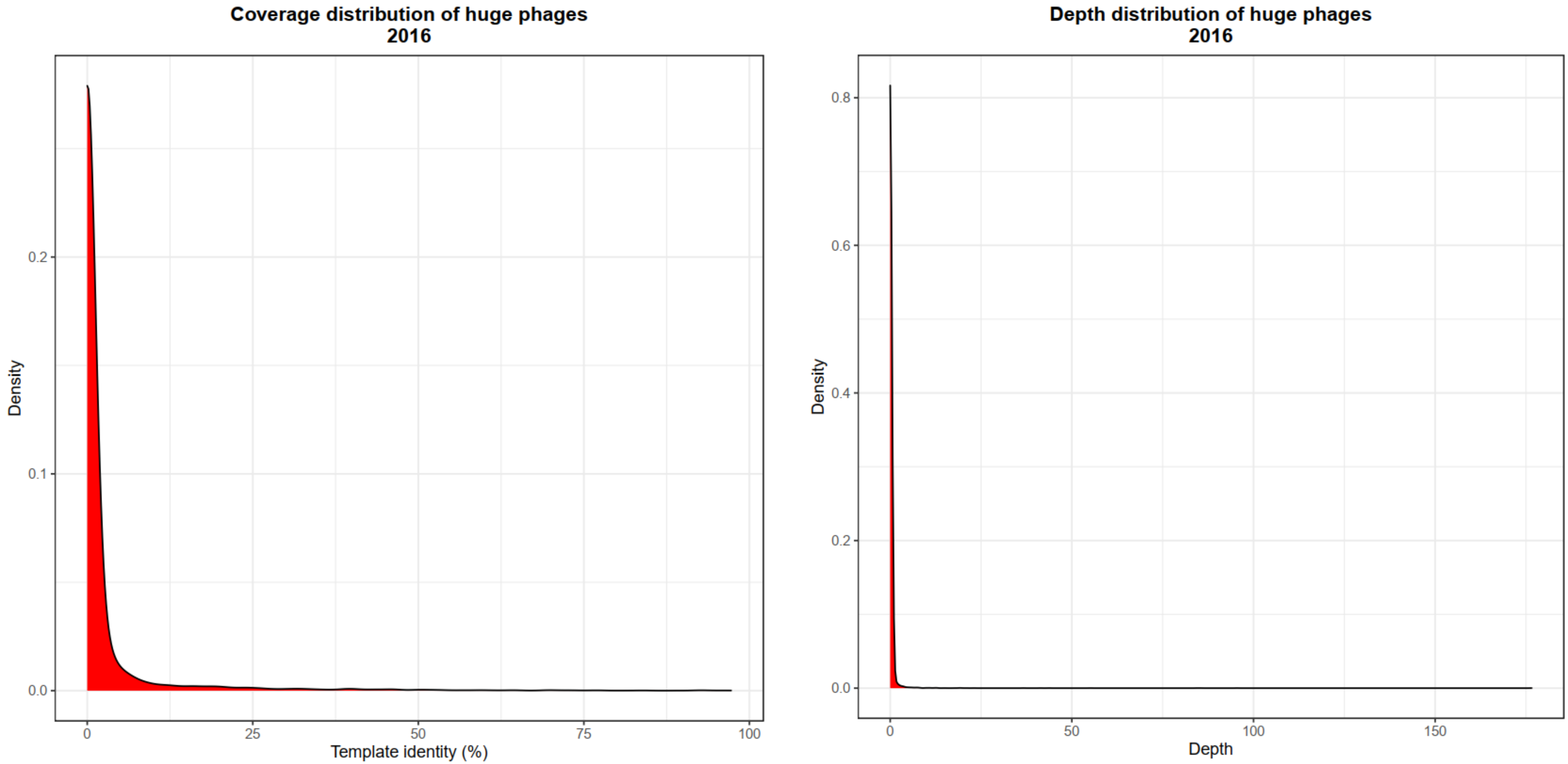

Supplementary Figure 3 Coverage (left) and depth (right) distributions for huge phages.

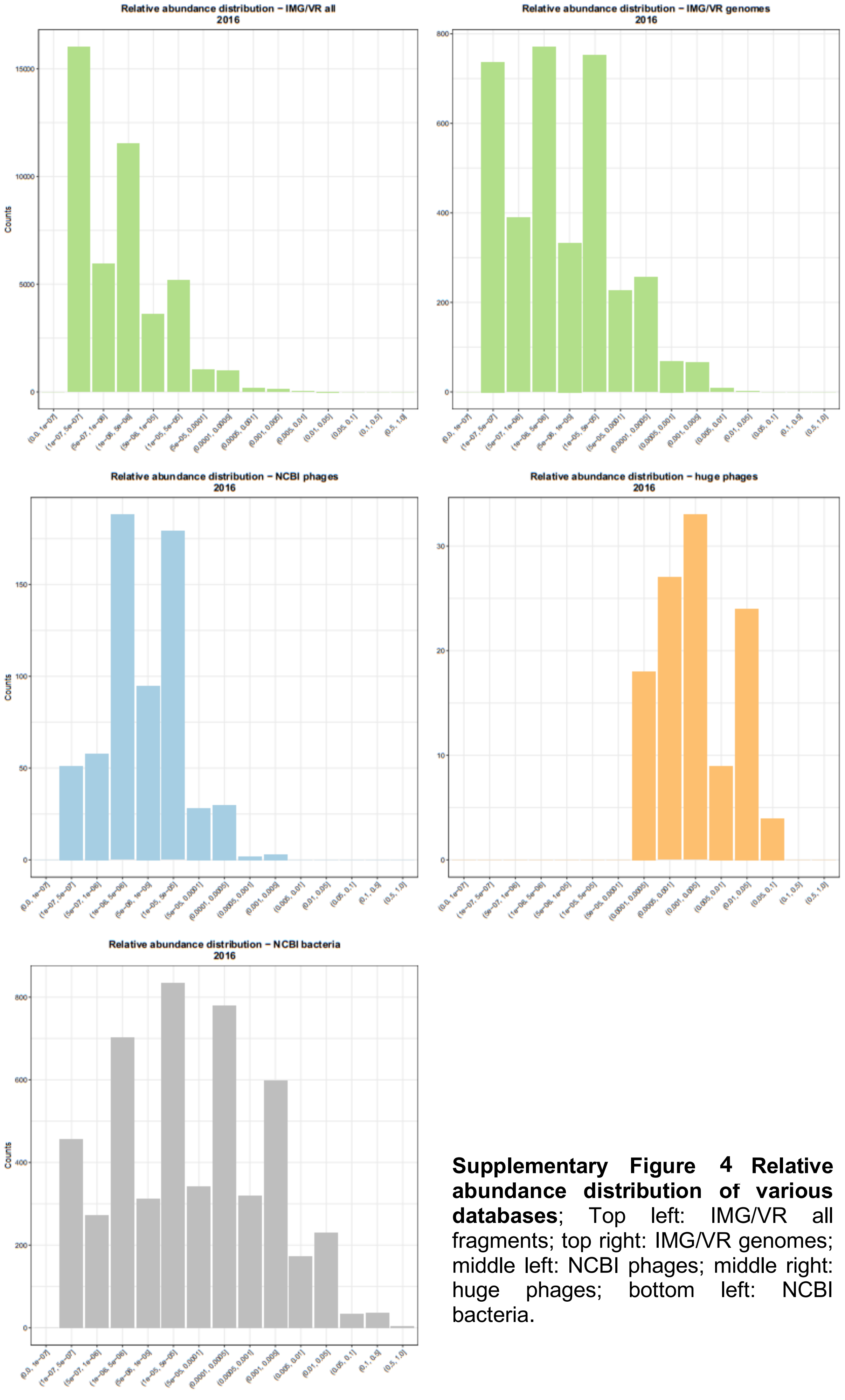

Supplementary Figure 4 Relative abundance distribution of various databases; Top left: IMG/VR all fragments; top right: IMG/VR genomes; middle left: NCBI phages; middle right: huge phages; bottom left: NCBI bacteria.

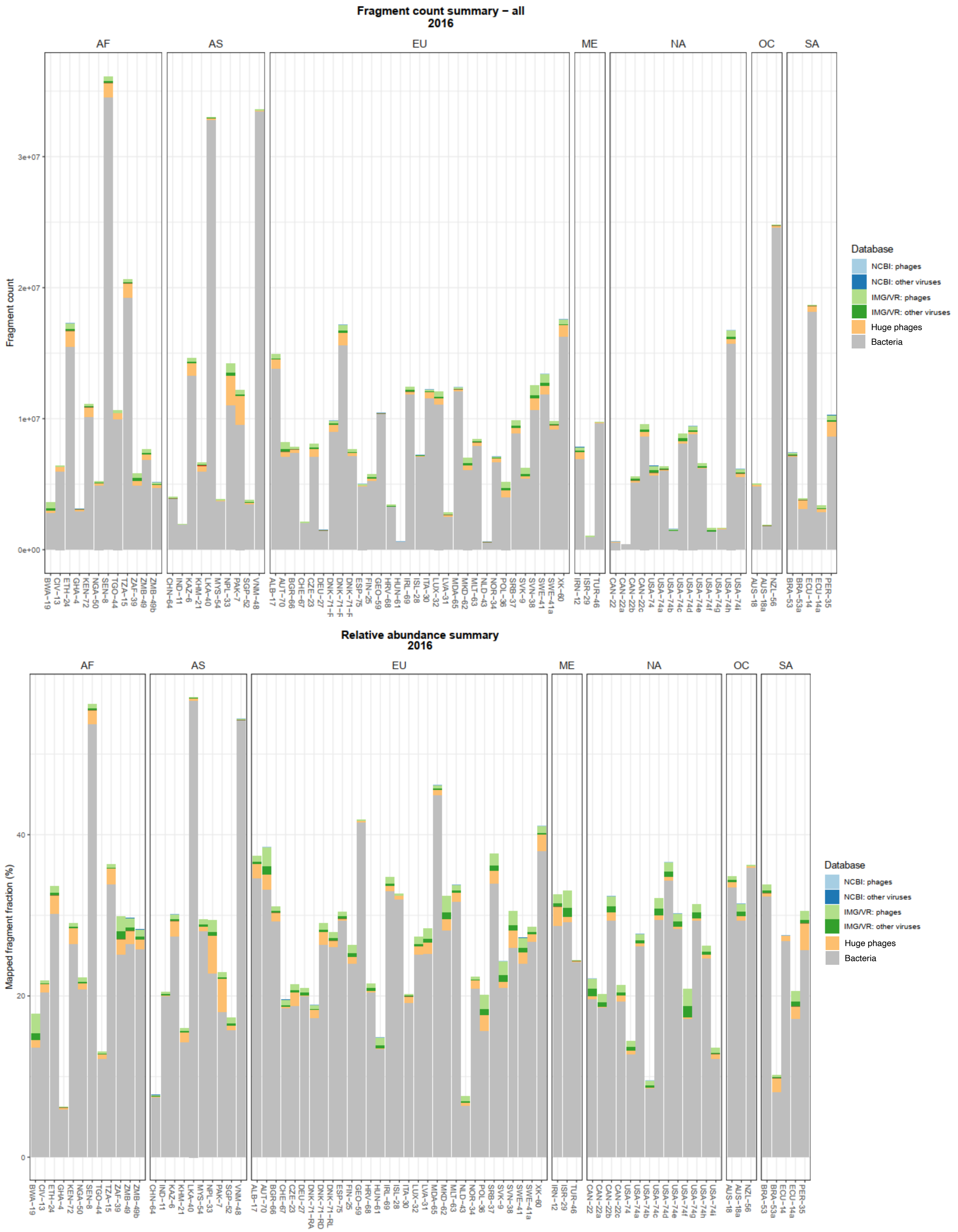

Supplementary Figure 5 Fragment mapping summary (top) and relative abundance summary (bottom) to bacterial and viral databases.
AF, Africa; AS, Asia; EU, Europe; ME, Middle East; NA, North America; OC, Oceania; SA, South America.

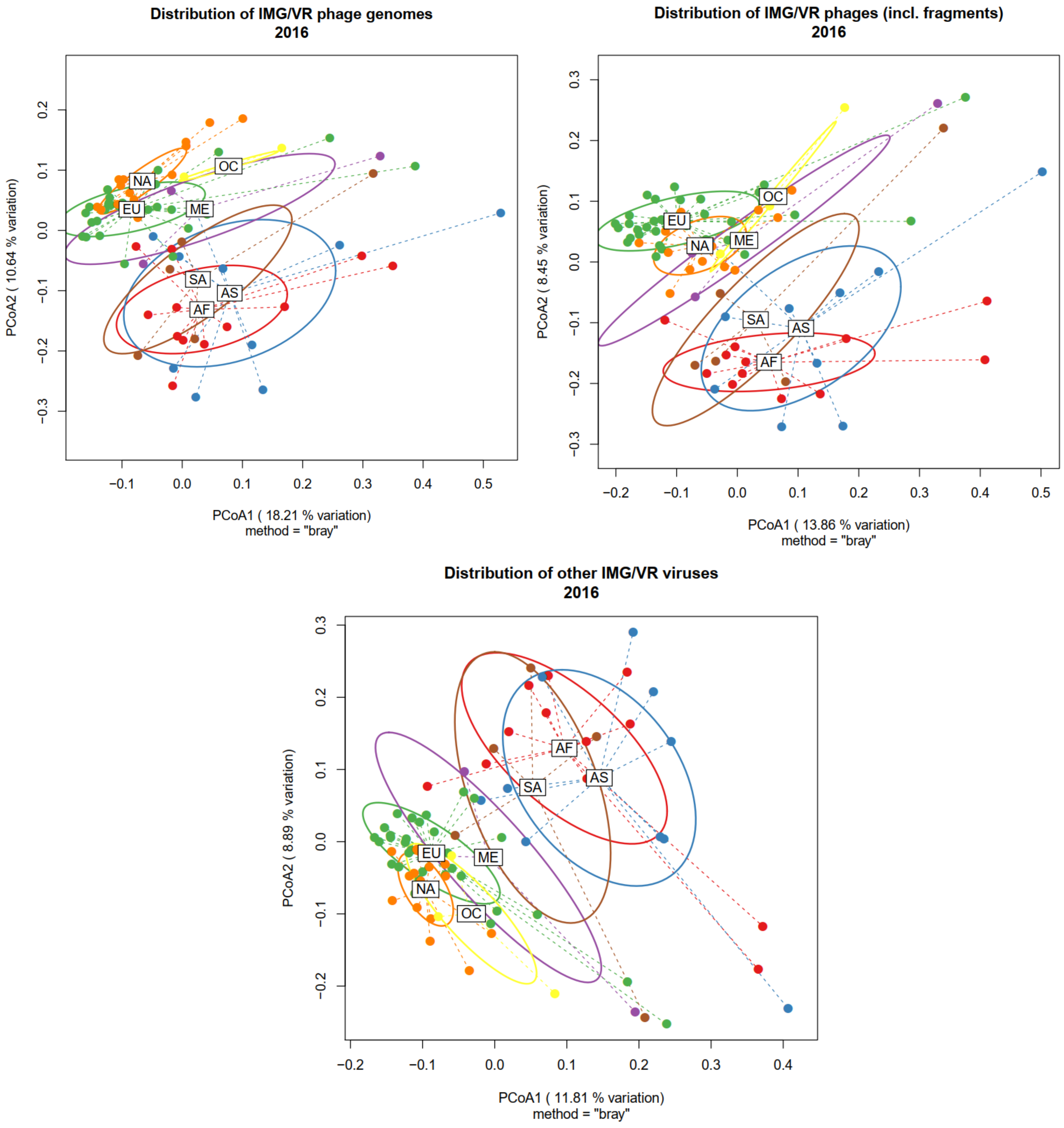

Supplementary Figure 6 PCoA of IMG/VR genomes (top left), all phages (incl. fragments, top right), and other viruses (bottom). Data stratified by region. Data is based on Bray-Curtis dissimilarities on Hellinger standardised relative abundances to account for phages of low-abundance. Variations are explained by X- and Y-axes (1st and 2nd coordinates).

AF, Africa; AS, Asia; EU, Europe; ME, Middle East; NA, North America; OC, Oceania; SA, South America.

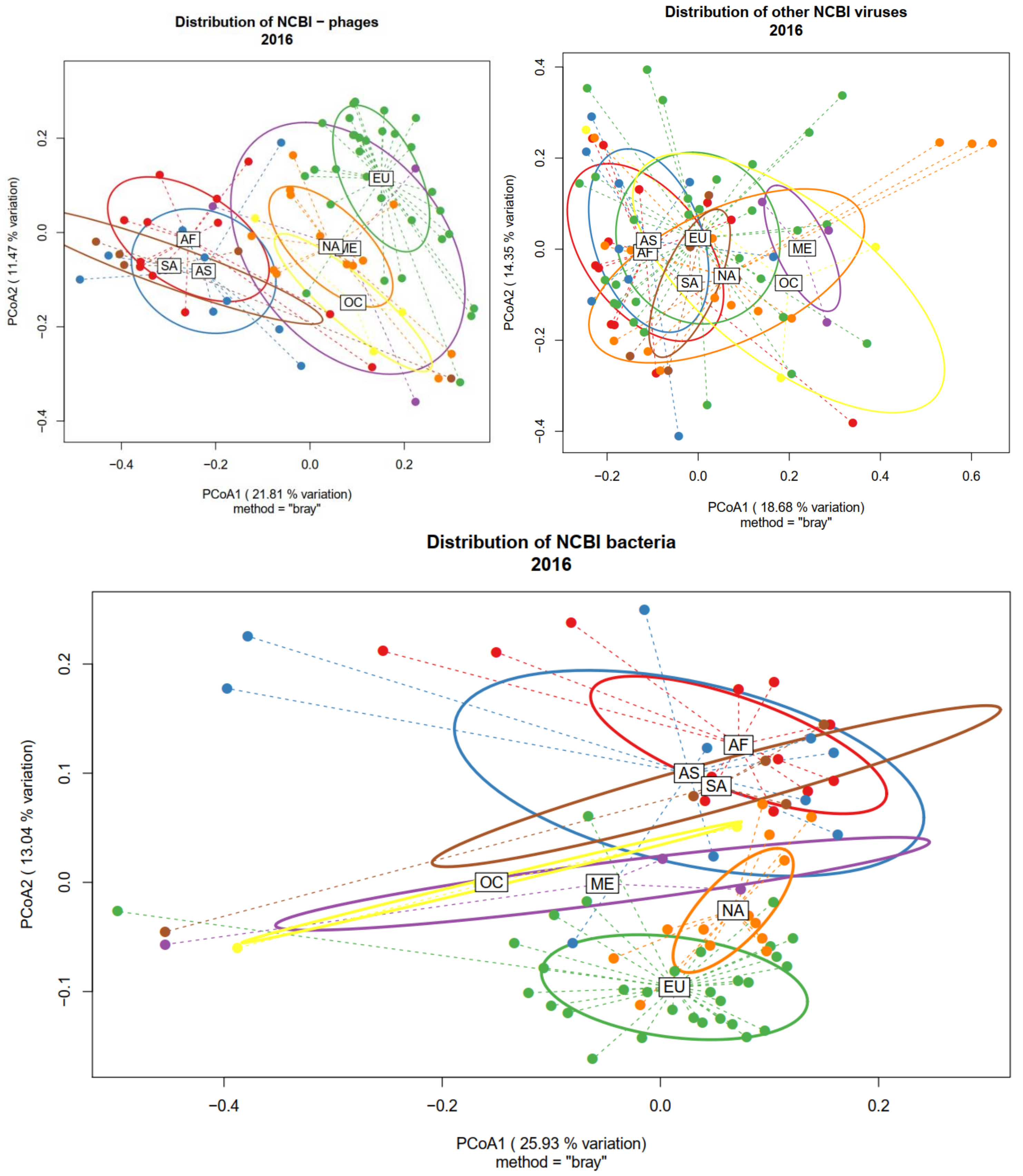

Supplementary Figure 7 PCoA of NCBI phages (top left), other viruses (top right), and bacteria (bottom).

Data stratified by region. Data is based on Bray-Curtis dissimilarities of Hellinger standardised relative abundances to account for phages of low-abundance. Variations are explained by X- and Y-axes (1st and 2nd coordinates).

AF, Africa; AS, Asia; EU, Europe; ME, Middle East; NA, North America; OC, Oceania; SA, South America.

Supplementary Figure 8 Relative abundances of top 15 NCBI phages per sample.

204 total phages. Colour intensity based on 0.5%-quantiles (deciles). Columns grouped by Bray-Curtis dissimilarities of Hellinger standardised relative abundances. Rows hierarchically clustered by Pearson correlation.

AF, Africa; AS, Asia; EU, Europe; ME, Middle East; NA, North America; OC, Oceania; SA, South America.

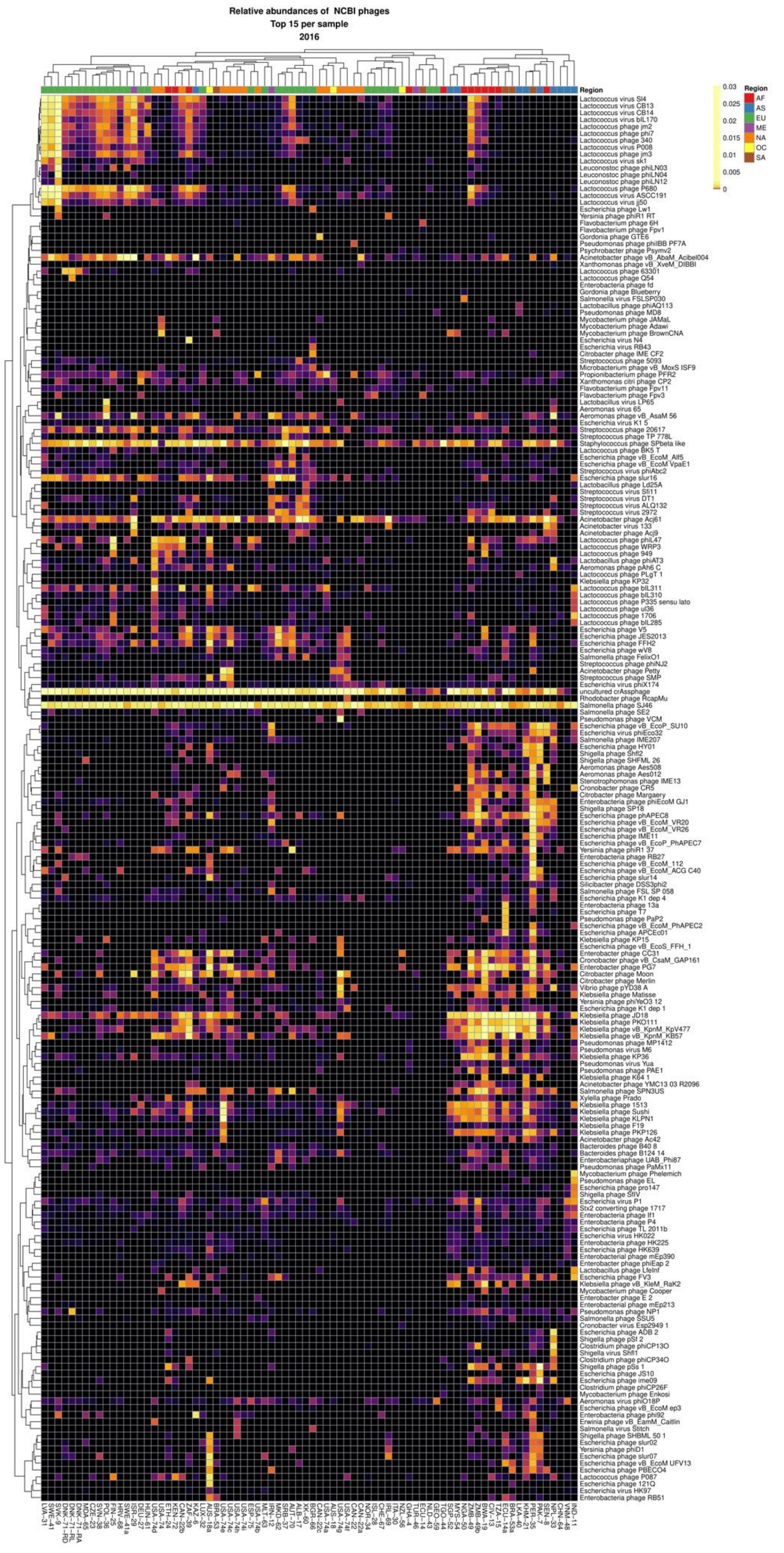

Supplementary Figure 9 Relative abundances of top 15 IMG/VR genomes.

173 total phages. Colour intensity based on 6.6%-quantiles. Columns clustered according to Bray-Curtis dissimilarities of Hellinger standardised relative abundances. Row hierarchically clustered according to their Pearson correlation.

AF, Africa; AS, Asia; EU, Europe; ME, Middle East; NA, North America; OC, Oceania; SA, South America.

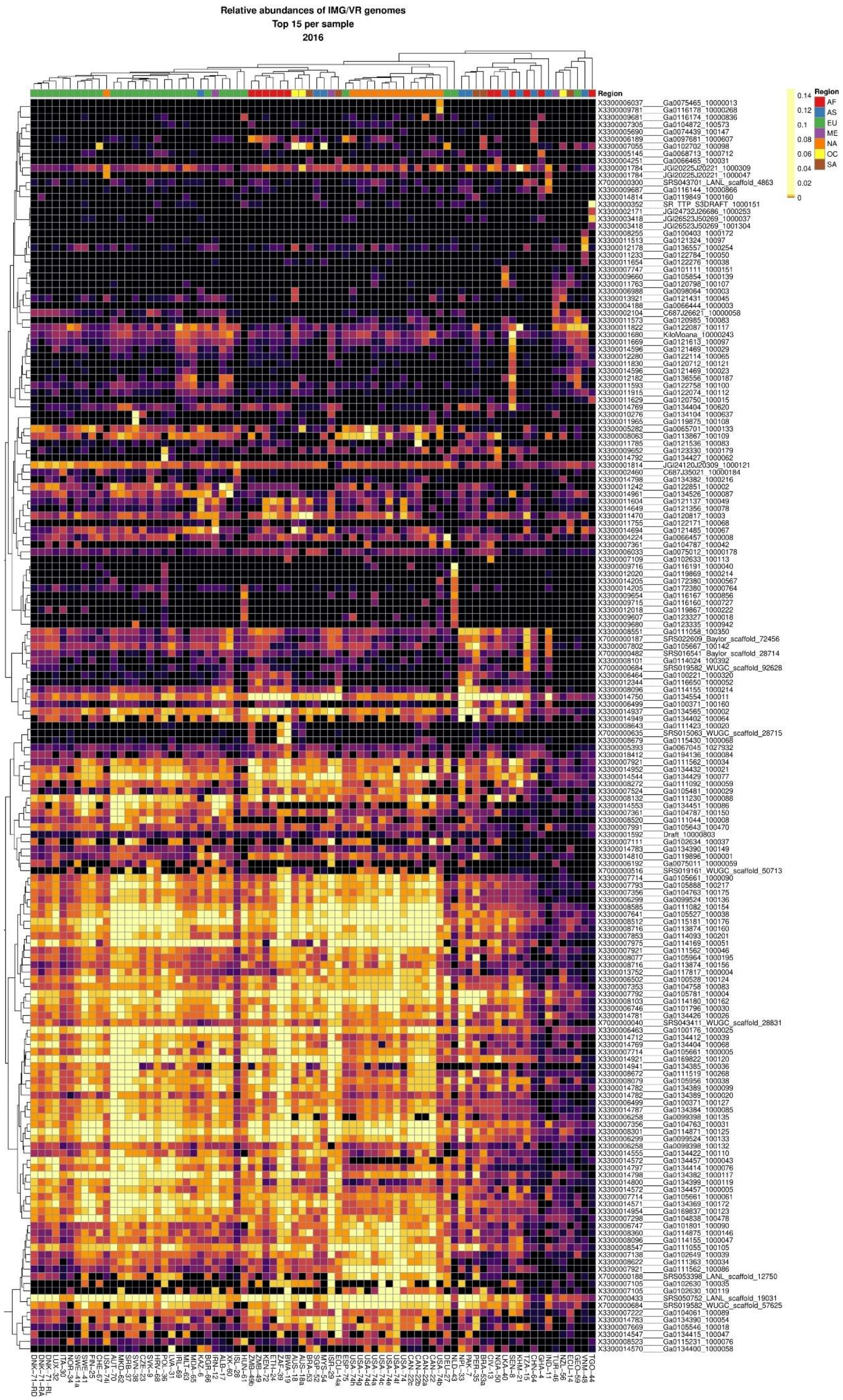

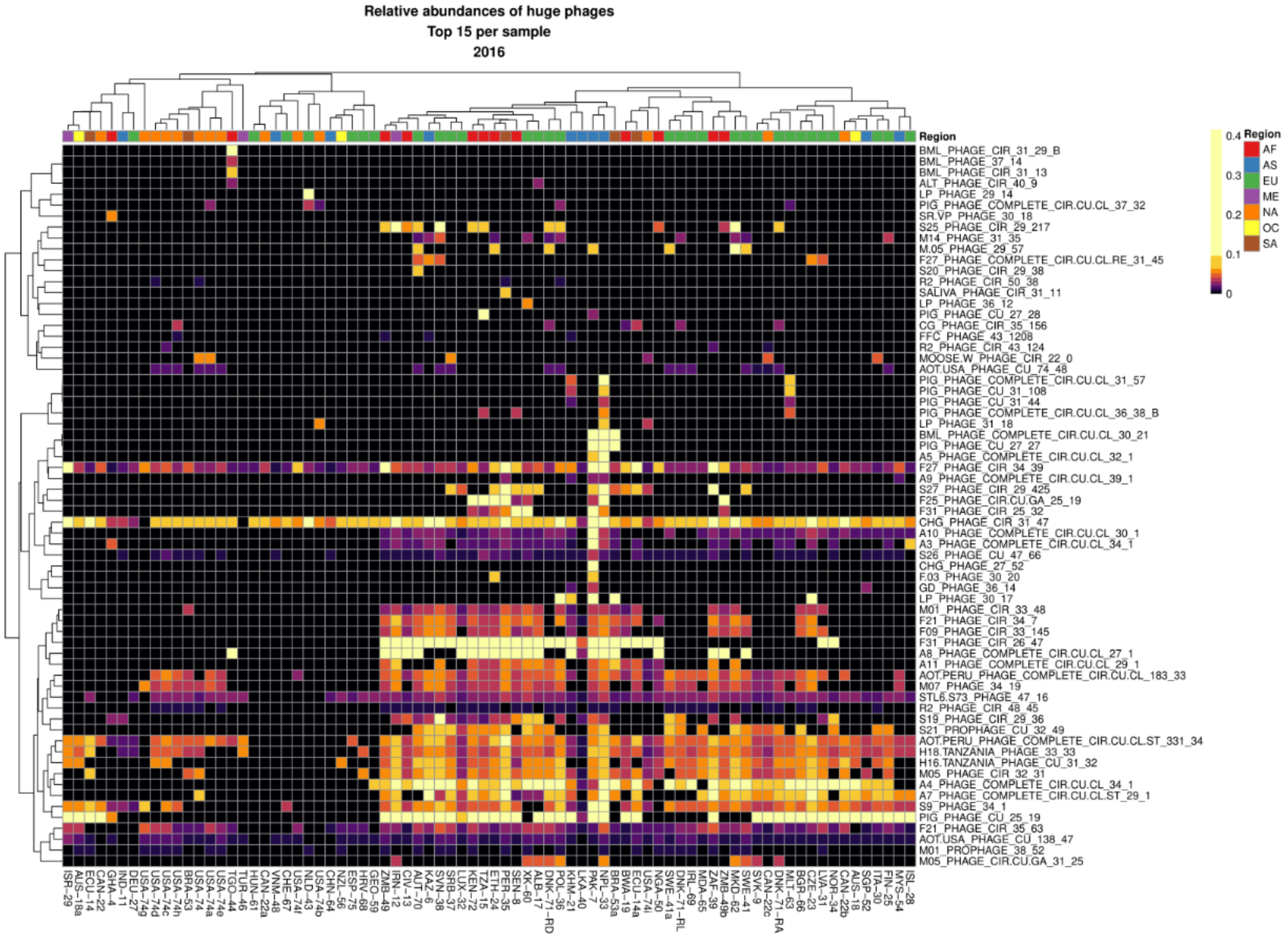

Supplementary Figure 10 Relative abundances for top 15 of huge phages per sample.
67 total phages. Colour intensity based on 3.3%-quantiles. Columns clustered according to Bray-Curtis dissimilarities of Hellinger standardised relative abundances. Rows hierarchically clustered according to Pearson correlation.
AF, Africa; AS, Asia; EU, Europe; ME, Middle East; NA, North America; OC, Oceania; SA, South America.

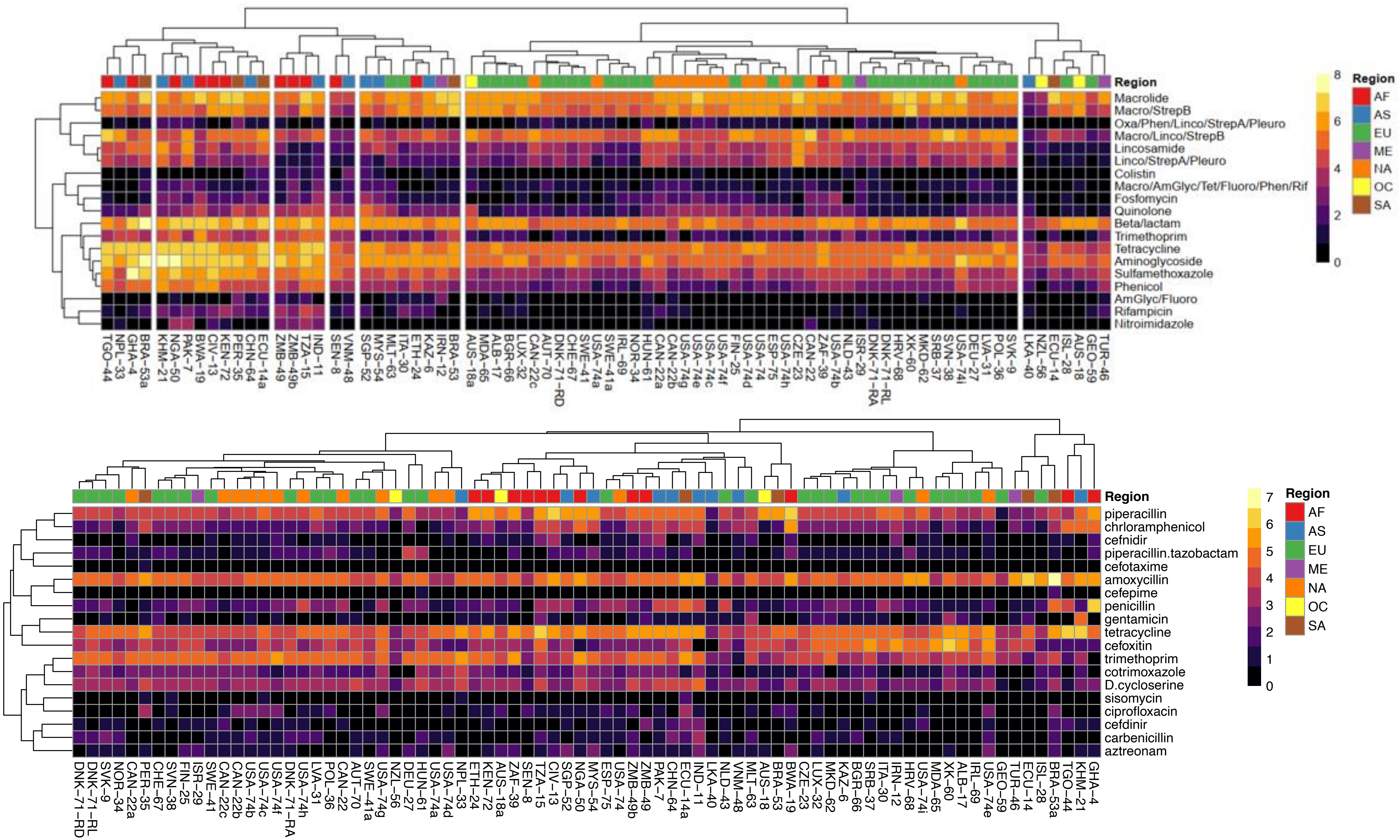

Supplementary Figure 11 Log-transformed FPKM of ARGs identified from ResFinder (top) and Functional Resistance (bottom). Amino, Aminoglycoside; Fluoro, Fluoroquinolone; Linco, Lincosamide; Macro, Macrolide; Oxa. Oxazolidinone; Phen, Phenicol; Pleuro, Pleuromutilin; Rif, Rifampicin; StreptoA, Streptogramin A; StreptoB, Streptogramin B; Tet, Tetracycline. AF, Africa; AS, Asia; EU, Europe; ME, Middle East; NA, North America; OC, Oceania; SA, South America

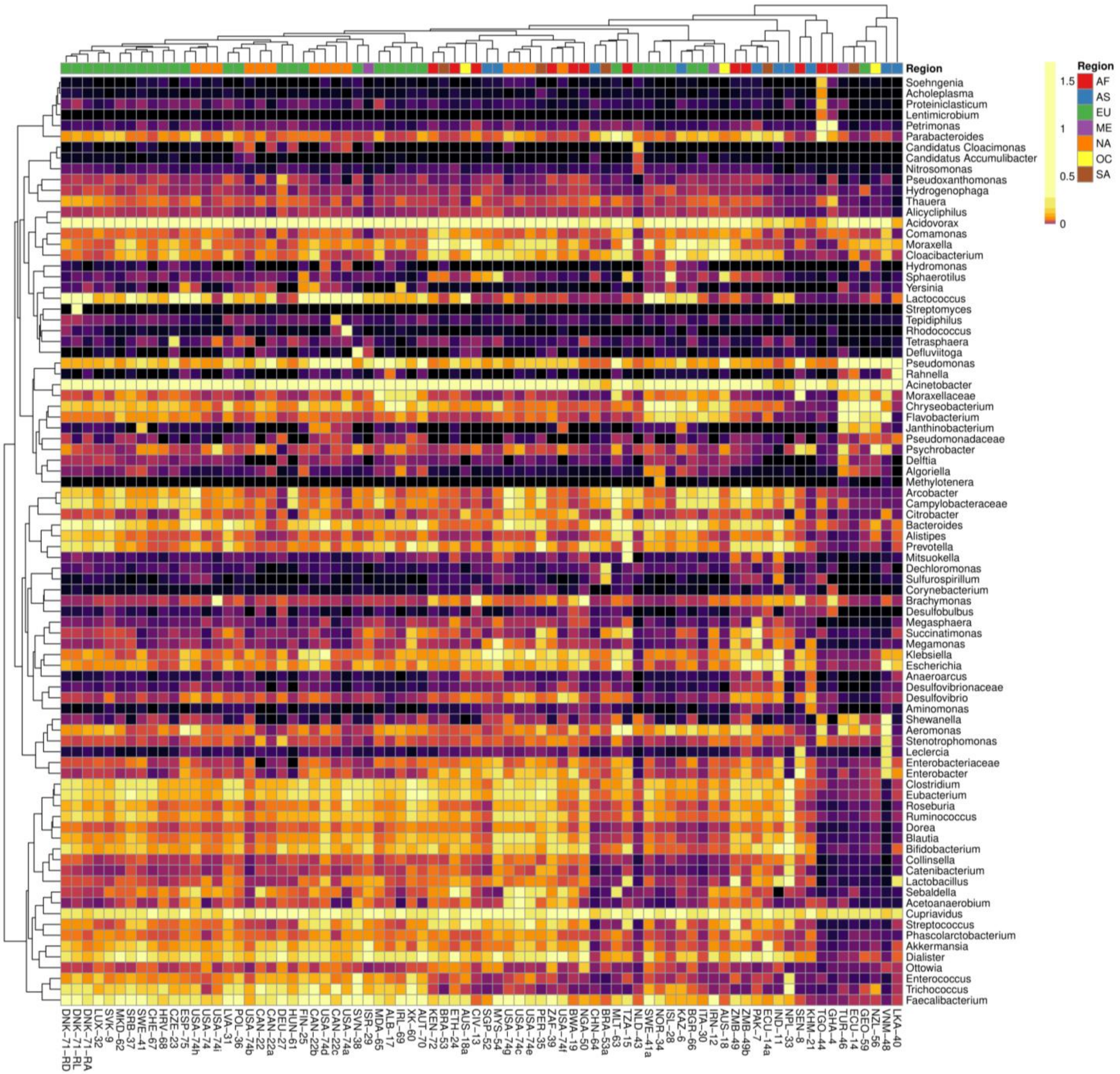

Supplementary Figure 12 Relative abundances of top 15 NCBI bacterial genera. Intensities based on 10% quantiles.
AF, Africa; AS, Asia; EU, Europe; ME, Middle East; NA, North America; OC, Oceania; SA, South America.
